# Abattoir Surveillance of Suspected Cystic Echinococcosis in Cattle: Insights From Molecular Analysis in a Hypoendemic Area

**DOI:** 10.1155/vmi/6674539

**Published:** 2026-10-14

**Authors:** M. Rega, G. Pupillo, M. Genchi, A. Parrinello, M. Conter, C. Loiudice, Laura L. Kramer, A. Vismarra, A. B. Casano, M. Fozzer

**Affiliations:** ^1^ Department of Veterinary Sciences University of Parma Strada Del Taglio 10 43126 Parma, Italy unipr.it; ^2^ Istituto Zooprofilattico Sperimentale Della Lombardia ed Emilia Romagna Via Pitagora, 2 42124 Reggio Emilia Italy; ^3^ Azienda Unità Sanitaria Locale (AUSL) Parma Via Giorgio Vasari 13A 43126 Parma Italy

**Keywords:** bovine, *Echinococcus granulosus*, laboratory analyses, northern Italy, postmortem inspection

## Abstract

Cystic echinococcosis (CE) is a zoonotic disease of global public health impact. In hypoendemic regions, the epidemiological role of cattle as intermediate hosts remains unclear, and routine abattoir‐based surveillance may overestimate prevalence due to diagnostic limitations. This study integrated a retrospective and prospective study of CE in cattle from the hypoendemic areas of Emilia‐Romagna and Lombardy regions (northern Italy) based on postmortem surveillance at slaughterhouses. Retrospective data were analyzed over four years (2021–2024), and the prospective study was performed in 2025, combining macroscopic inspection with anatomopathological and molecular analyses to evaluate diagnostic accuracy. The retrospective analysis revealed a low mean prevalence of suspected CE (0.32%), while prospective evaluation showed an apparent prevalence of 0.08% based on macroscopic inspection. However, molecular analyses revealed the absence of *Echinococcus granulosus* and *E. multilocularis* DNA, while 50% of isolated lesions were caused by *Taenia hydatigena*. The integration of molecular diagnostics with in‐depth anatomopathological evaluation improves diagnostic accuracy, reduces misclassification, and allows risk‐targeted control strategies. Prioritizing confirmatory diagnostics and targeted interventions is recommended in a One Health approach.

## 1. Introduction

Ecological, geopolitical, and economic factors favor the emergence of infectious diseases worldwide. The increasing movement of people and animals facilitates the spread of pathogens across geographical boundaries. More than 60% of emerging infectious diseases reported globally originate from animals, posing major challenges to both human and animal health. Addressing the impact of emerging zoonotic pathogens, as well as neglected and endemic diseases, requires adequate resources and coordinated surveillance within a One Health framework [1].

Cystic echinococcosis (CE) is a globally distributed zoonotic disease that poses significant public health concerns. The disease is a considerable burden on human health, with an estimated 184,000 disability‐adjusted life years (DALYs) lost annually. More than one million people are estimated to be infected worldwide at any given time [2]. In the European Union, 984 confirmed human cases of echinococcosis were reported in 2024 (0.22 cases per 100,000 population), representing a 19.1% increase compared with 2023. Among cases with species identification, *Echinococcus granulosus* sensu lato, the causative agent of CE, accounted for 62.9% of infections, while *E. multilocularis* accounted for the remaining 37.1%. CE also causes substantial economic loss in livestock production due to poor growth rates, reduced milk yield, decreased carcass weight, and organ condemnation at slaughterhouse [3].

*E*. *granulosus* is a tapeworm whose adult stage inhabits the small intestine of canines as definitive hosts [4]. When intermediate hosts, mainly herbivores, ingest parasite eggs, hydatid cysts develop in internal organs, primarily liver and lungs. These cysts are fluid‐filled, unilocular structures composed of a germinal layer, a laminated layer, and an outer host‐derived adventitial layer, and they produce protoscoleces infective to dogs. In humans, clinical manifestations vary depending on cyst size, number, growth rate, and location, ranging from asymptomatic to severe disease [3].

In livestock, CE is typically detected at slaughter, and data derived from official meat inspection are commonly used to estimate CE prevalence in cattle, sheep, and pigs [5]. Although serology can also be employed to detect infection in livestock, necropsy remains the diagnostic gold standard and is widely used as a reference method for evaluating the sensitivity and specificity of immunodiagnostic tests [4].

In accordance with European Directive 2003/99/EC [6], active monitoring for CE is required at the most appropriate stage of the food chain in all European Member States. Regulation (UE) No 625/2017 [7] assigns official veterinarians at slaughterhouses the responsibility of performing visual inspection of offal, complemented by standardized incisions, a procedure considered suitable for the detection of CE. When positive animals are identified, cases are reported to the competent local authority, which subsequently conducts an epidemiological investigation at the farm (s) of origin to enact control measures, including anthelmintic treatment of farm dogs [8].

The effectiveness of this strategy is questionable, since the definitive source of infection is rarely demonstrated. Fecal samples from farm dogs are seldom analyzed for the presence of taeniid eggs, and molecular analyses are not routinely performed to confirm the etiological agent responsible for the detected lesions. Consequently, treatment of farm dogs may be unnecessary or ineffective [8]. Nevertheless, the epidemiological role of cattle in CE transmission remains controversial, as these large ruminants are frequently found infected with non–host‐specific *Echinococcus* species [9].

CE requires a multisectoral approach and increasing attention to the One Health framework, particularly in complex transmission systems involving humans, animals, and the environment [1]. This is also essential for hypoendemic areas to maintain the favorable epidemiological trend of the disease. Previous studies have reported the incidental detection of CE in dairy cattle raised in intensive farming systems without access to pasture, highlighting unexpected patterns of infection [9]. Historically, northern Italy has been considered a hypoendemic area for bovine CE, with prevalence generally lower than 1%. In Emilia‐Romagna, CE prevalence in cattle was estimated at only 0.41%–0.54%, with a localized cluster (1.4%) reported in the northwestern area of Reggio Emilia (RE) province [10]. Recent national data confirm that the burden of human CE in Italy remains concentrated in the southern regions and the two major islands, whereas northern regions continue to account for only a small proportion of human cases [11, 12].

The present study reports results of a retrospective analysis of visually diagnosed CE in dairy cattle from the Emilia‐Romagna and Lombardy regions (northern Italy) identified at slaughter during routine postmortem inspections between 2020 and 2024. In addition, a prospective study was carried out in 2025 to confirm the nature of the detected cysts through detailed anatomopathological examination and molecular diagnostic analyses. This approach enabled the validation of prevalence estimates obtained through visual inspection in low‐endemic areas and allowed the characterization of the parasite genotype.

## 2. Materials and Methods

### 2.1. Retrospective Study

A retrospective observational study was conducted to evaluate the prevalence of cystic lesions compatible with *E*. *granulosus* in intensively‐farmed dairy cattle from a bovine slaughterhouse located in Emilia‐Romagna region. The study period covered January 2021 to September 2024. Data were collected through analysis of official slaughterhouse records of the Competent Authority during routine postmortem inspections.

All cattle bred in the Emilia‐Romagna and Lombardy regions (Northern Italy) slaughtered during the study period and recorded in the official registers were included in the study. Postmortem inspections were performed by official veterinarians in accordance with current European legislation. Lesions were classified as hydatid cysts based on macroscopic evaluation, including well‐defined fluid‐filled cysts, without parasitological or molecular confirmation.

For each animal, the following data were collected when available: age at slaughter, sex, geographical origin, presence of cystic lesions compatible with *E. granulosus*, and anatomical localization of the lesions (e.g., liver, lungs, or other organs). Animals with incomplete or missing essential data were excluded from specific analyses when appropriate.

#### 2.1.1. Statistical Analyses

Descriptive statistics was used to summarize the distribution of cattle with lesions compatible with CE according to year and province of origin. Apparent prevalence was calculated as the number of positive animals divided by the total number of cattle slaughtered within each year and province.

Differences in the probability of lesion detection among years and provinces were assessed using a generalized linear model with a binomial error distribution and logit link function. The grouped number of positive and negative animals was used as the response variable, while year and province of origin were included as categorical explanatory variables. The overall significance of each factor was evaluated by likelihood‐ratio chi‐square tests through comparison of nested models. Adjusted odds ratios (ORs), with corresponding 95% confidence intervals (95% CIs), were calculated using 2021 as the reference year. Model dispersion was evaluated using the ratio between the Pearson chi‐square statistic and the residual degrees of freedom. Statistical significance was set at a two‐sided *P* value < 0.05. Analyses were performed using Python Version 3.13.5 and the Statsmodels package Version 0.14.6.

### 2.2. Prospective Study

A prospective study was conducted between October 2024 and March 2025 in the same slaughterhouse where the retrospective study was conducted, with a sample size of 17,541 animals. Animals were eligible for inclusion if they were from intensive farms located in Lombardy or Emilia‐Romagna regions. All carcasses underwent routine postmortem inspection performed by the official veterinarian in accordance with Regulation (EU) 2017/625 [7] on official controls and Commission Implementing Regulation (EU) 2019/627 [14], which lays down the uniform practical arrangements for ante‐ and postmortem inspection of products of animal origin intended for human consumption. Postmortem examination included systematic visual inspection of the carcass and edible offal, with palpation and incision performed whenever required according to the risk‐based approach established by the legislation. For each animal, the following data were collected: age at slaughter, sex, geographical origin, presence of cystic lesions compatible with *E. granulosus*, and anatomical localization of the lesions (e.g., liver, lungs, or other organs).

Suspected hydatid cysts were excised together with a margin of approximately 2 cm of surrounding tissue to preserve lesion integrity and avoid contamination. Each specimen was individually placed in a sterile plastic bag, appropriately labeled with a unique identification code, and transported under refrigerated conditions and according to biosafety procedures to the Parasitology Laboratory of the University of Parma for further analyses.

#### 2.2.1. Anatomopathological Analyses

Lesions were incised and inspected for typical morphological features of CE and the eventual presence of protoscolices by microscopic examination of cystic liquid to determine fertile versus sterile cysts. Lesions were considered compatible with CE based on their gross appearance, including cystic lesions with fluid content, whitish internal membranes and/or calcified or degenerated lesions [13, 14]. After gross examination, lesions were isolated from surrounding tissues and collected for molecular analyses, including parasite genotyping.

#### 2.2.2. Molecular Analysis

DNA was extracted from cyst fragments and fluids. For cysts, 25 mg of tissue was homogenized with 300 µL of PBS in a Tissue homogenizer (GeneReady homogenizer, Life Real, China) (4 cycles of 1 min at 6.5 m/s), followed by an overnight incubation at 56°C (Thermomixer F2.0, Eppendorf, Germany) with 180 µL of Digestion buffer and 20 µL proteinase K. Then, the manufactures’ instructions were followed (PureLink Genomic DNA Mini Kit, Invitrogen, USA), and DNA was eluted in a final volume of 50 µL.

A multiplex PCR was carried out on DNA extracted according to Trachsel et al. [10], with slight modifications, using one common reverse primer and three target‐specific forward primers. This protocol is able to discriminate among *Taenia* spp., *E. granulosus* and *E. multilocularis* using the following primers: UnivSr, TaeSf, EgS2f, EmN2f, and EmNr at a final concentration of 0.2 pmol/μL each and 0.4 pmol/μL for UnivSr. DNA extracted from a confirmed *Echinococcus granulosus* sensu lato (G1 genotype) ovine hydatid cyst was included as a positive control in each PCR run, while nuclease‐free water was used as the negative control. To monitor PCR inhibition, an internal amplification control (IC) was included in each reaction according to the validated laboratory protocol. All samples were analyzed in duplicate to confirm the reproducibility of the molecular results. The amplification protocol foresaw an initial denaturation at 95°C for 5 min and 40 cycles as follow: 95°C for 30s, 52°C 30 s, 72°C 45 s with a final elongation step at 72°C for 8 min. PCR products (*Taenia* spp.: 267 bp, *E. granulosus*: 116 bp, *E. multilocularis*: 394 bp) were run on agarose gel at 2% and positive bands were cut, purified with a commercial kit (Wizard SV Gel and PCR Clean‐Up, Promega, USA), and sent to Istituto Zooprofilattico della Lombardia ed Emilia Romagna (IZSLER) for genome sequencing (Sanger method). Sequences were analyzed with MEGA6.0 software, aligned with ClustalW, and compared with published sequences from GenBank by BLAST.

## 3. Results

### 3.1. Retrospective Study

The study analyzed slaughterhouse records from a total of 129,957 cattle slaughtered in a bovine facility in Emilia‐Romagna, focusing on lesions registered as CE by the official veterinarian between 2021 and 2024. In particular, 28,962 animals were included in 2021, 33,721 in 2022, 33,090 animals in 2023, and 34,184 in 2024. Their geographical distribution is reported in (Figure 1). All the animals were female. Cattle came from several provinces of Emilia‐Romagna and Lombardy: RE, Piacenza (PC), Bologna (BO), Parma (PR), Ferrara (FE), Ravenna (RA), Forlì‐Cesena (FC), Rimini (RN), Lodi (LO), Mantova (MN), Cremona (CR), Milano (MI), Bergamo (BG), Brescia (BS), Como (CO), Pavia (PV), and Varese (VA).

**FIGURE 1:**
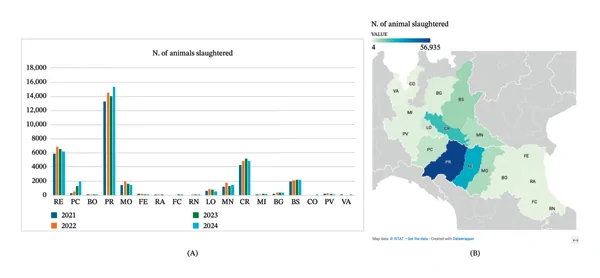
Distribution by province of the animals included in the retrospective study. (A) Number of animal slaughtered per province per year. (B) Geographical distribution of the overall animal slaughtered per province.

Over the four‐year period, the overall mean prevalence of disease was 0.32%. Specifically, prevalence was 0.24% (70/28,962; 95% CI: 0.19–0.31) in 2021, 0.21% (71/33,721; 95% CI: 0.17–0.27) in 2022, 0.45% (150/33,090; 95% CI: 0.39–0.53) in 2023%, and 0.38% (131/34,184; 95% CI: 0.32–0.45) in 2024. The prevalence per province per year is reported in Figure 2. The provinces with the highest mean prevalence of bovine with CE lesions were BO (1.30%), PC (0.43%), and BG (0.39%). The provinces of LO, MN, and CR showed an increasing trend in disease prevalence, followed by a plateau or slight decrease in 2024. In contrast, FE, RA, FC, RN, MI, CO, PV, and VA had no positive animals during the study period; however, these provinces had a very low number of slaughtered animals per year. The province of BO exhibited the highest prevalence over the four years with a marked decline from 2021 to 2024. The provinces of RE, PR, MO, BG, and BS showed a slight variable pattern throughout the study period.

**FIGURE 2:**
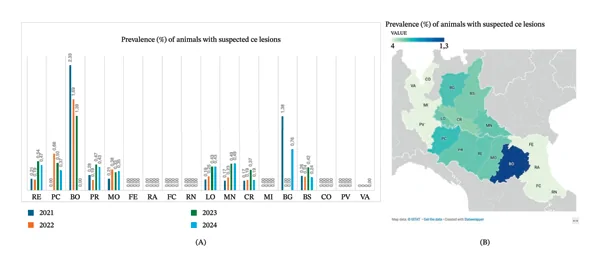
Prevalence (%) by province of animals with lesions compatible with EC. (A) Prevalence of animals with suspected CE lesions per province per year. (B) Geographical distribution of the overall animals with suspected CE lesions per province.

In the binomial regression model, year was significantly associated with the probability of detection of CE lesions (likelihood‐ratio χ^2^ = 40.07, df = 3, *p* < 0.001). After adjustment for province of origin, the odds of positivity were higher in 2023 (OR = 1.88; 95% CI: 1.41–2.49; *p* < 0.001) and 2024 (OR = 1.57; 95% CI: 1.17–2.10; *p* = 0.002) than in 2021. No significant difference was observed between 2022 and 2021 (OR = 0.87; 95% CI: 0.62–1.21; *p* = 0.400). Conversely, province of origin was not significantly associated with positivity after adjustment for year (likelihood‐ratio χ^2^ = 19.88, df = 17, *p* = 0.280).

The majority of slaughtered animals were aged between 24 and 59 months. Most animals with lesions compatible with CE were aged between 60 and 95 months across the study period (Figure 3). In most cases, the localization of the suspected lesions was not specified in the slaughter records. When reported, hepatic lesions were more frequent than pulmonary lesions.

**FIGURE 3:**
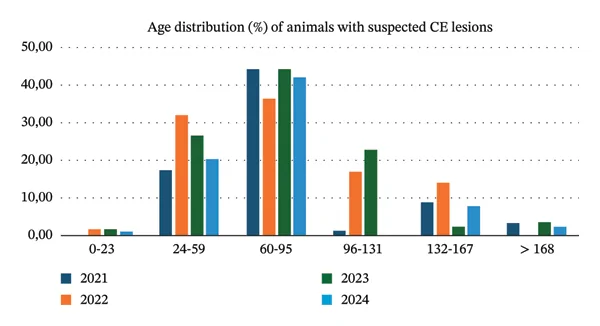
Age distribution of animals with suspected CE lesion. Percentages are calculated on the total number of diseased animals per year.

### 3.2. Prospective Study

During the selected sampling period (October 2024 to March 2025), a total of 17,541 female cattle were slaughtered at the selected abattoir. The majority of animals originated from intensive farms located in the province of PR (45%) and RE (18%), as detailed in (Figure 4).

**FIGURE 4:**
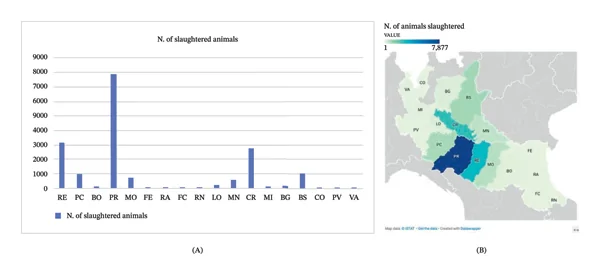
Distribution by province of the animals included in the prospective study. (A) Number of animal slaughtered per province. (B) Geographical distribution of animals slaughtered per province.

Following postmortem inspection, 16 hepatic cystic lesions were collected from 16 female cattle. No cystic lesions were macroscopically detected in the lungs or other viscera. The suspected prevalence of *Echinococcus granulosus sensu lato* was 0.09% (16/17,541; 95% CI 0.06–0.15) (Figure 5).

**FIGURE 5:**
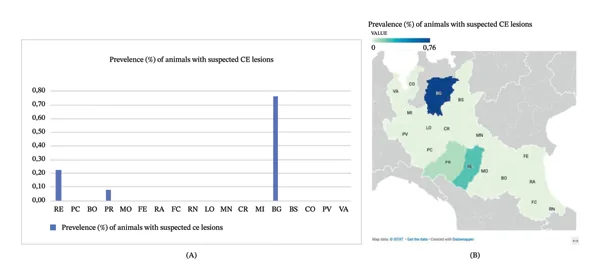
Prevalence (%) by province of animals with lesions compatible with EC. (A) Prevalence of animals with suspected CE lesion per province. (B) Geographical distribution of animals with suspected CE lesion per province.

Anatomopathological examinations performed at the University of Parma revealed heterogeneous morphology among the evaluated lesions. None of the cysts showed macroscopic evidence of cystic fluid or hydatid sand. The lesions were subcapsular, and, in some cases, the cyst wall appeared damaged due to mechanical trauma during viscera handling at slaughter. Larger lesions extend slightly into the hepatic parenchyma (Figure 6).

**FIGURE 6:**
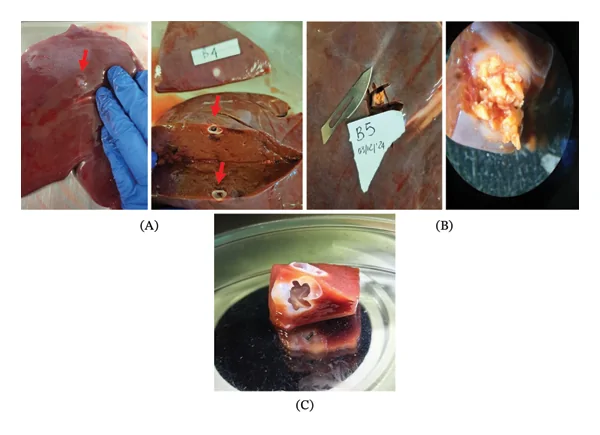
Different shades of the same macroscopical lesion. Some livers showed small lesions (less than 0.5 cm in diameter) with fluid (A) or calcified content (B), appearing firm and chalky on section, with the presence of white‐yellowish granular material. Other livers exhibited lesions larger than 1 cm in diameter, containing fluid, and some of these were multiloculated (C).

Most samples were characterized by a single lesion, while multifocal lesions were observed only in rare cases. Of the 16 cystic lesions, 2 were not morphologically attributable to *Echinococcus granulosus* and were therefore excluded from further analysis and the suspected prevalence decreased from 0.09% to 0.08% (95% CI: 0.05–0.13). Of the 14 morphologically confirmed hydatid cysts, 6/14 were found in animals from the province of PR, 7/14 from RE, and 1/14 from BG. The majority of suspected positive animals were aged between 60 and 95 months (8 animals). The others were aged 96–131 months (4 animals), one aged 24–59 months, and one aged 132–167 months.

Molecular analysis by PCR showed that all samples were negative for *Echinococcus granulosus* and *Echinococcus multilocularis,* while 7/14 (50%) were positive for *Taenia* spp. Sequencing revealed that they all belonged to *Taenia hydatigena* species, with 96% nucleotide identity to reference sequences. Among these, one lesion was multilocular, two were unilocular, and four were calcified. To conclude, in the perspective study, the final prevalence of *Echinococcus granulosus* was 0%.

## 4. Discussion

This study provides an integrated retrospective and prospective evaluation of CE surveillance in cattle slaughtered in a hypoendemic area of Northern Italy, combining abattoir inspection data with anatomopathological and molecular analyses.

The retrospective analysis revealed a low overall mean prevalence of suspected CE (0.32%) over the four‐year period, consistent with previous reports from Northern Italy and other hypoendemic regions [14–16]. This pattern is compatible with incidental infections and aligns with previous observations questioning the epidemiological role of cattle in maintaining the life cycle of *Echinococcus granulosus*, particularly in intensive dairy farming systems [8, 9]. In these systems, cattle are more likely accidental intermediate hosts than effective reservoirs. Consistent with the retrospective findings, positive animals often originated from the same provinces, reinforcing the concept that CE is closely linked to farm‐level management practices. When lesion localization was reported, hepatic involvement was more frequent than pulmonary involvement. This finding is in line with the classical hypothesis that oncospheres migrate primarily via the portal system, making the liver the first and most affected organ [17]. Similar organ distributions have been reported in both endemic and hypoendemic areas [18].

As expected, all animals involved in the study were female, reflecting the slaughter of end‐of‐career dairy cows. While sex is, therefore, not epidemiologically informative, age may be more relevant. In hypoendemic areas with low infection pressure, cystic lesions are more likely to be detected in older animals due to the chronicity of the disease [14]. A substantial proportion of the lesions identified were small and calcified, a morphological feature suggestive of host‐mediated cyst degeneration resulting from an effective immune response [19]. Although the biological mechanisms underlying cyst calcification remain incompletely understood, these lesions are often challenging to distinguish macroscopically and are therefore routinely condemned as a precautionary measure during meat inspection [7, 20]. However, in accordance with the results of the study, they may also have alternative etiologies, including inflammatory, infectious (both bacterial and/or viral origin), parasitic (*Taenia hydatigena*), or traumatic origins [19]. The prospective component of the study provided crucial insights into the reliability of abattoir‐based surveillance.

Although the apparent prevalence based on macroscopic inspection was 0.09%, subsequent confirmatory analyses did not support any diagnosis of CE. Histopathological examination excluded two suspected lesions, and molecular analyses detected neither *Echinococcus granulosus* sensu lato nor *Echinococcus multilocularis*. Instead, *Taenia* spp. DNA was identified in 50% of the examined lesions and was subsequently confirmed as *Taenia hydatigena* by genome sequencing. Consequently, the confirmed prevalence of CE in the study population was zero.

Not all PCR‐negative lesions could be definitively characterized. Besides *Taenia hydatigena*, cystic lesions detected in the lungs and liver of cattle may also be associated with other pathological conditions, including abscesses, chronic fibrotic lesions, calcified parasitic remnants, and traumatic lesions. Lungworms may also produce nodular pulmonary lesions that can be confused with parasitic cysts during gross inspection [21, 22].

These findings reinforce previous reports highlighting that cystic lesions detected at slaughter are not always parasitic in origin and that macroscopic inspection alone may lead to misclassification, especially in low‐prevalence settings [4, 8, 23]. Moreover, Wilson et al. (2019) demonstrated that, despite high specificity of routine carcass inspection, its limited sensitivity may lead to an underestimation of the true prevalence of CE because infected animals may remain undetected. However, diagnostic challenges have been reported in other European contexts, where molecular confirmation significantly reduced the number of true CE cases [9].

Furthermore, routine meat inspection is known to have limited sensitivity for detecting all CE lesions, particularly small, early‐stage or atypically located cysts. Therefore, slaughterhouse‐based surveillance is likely to underestimate the true prevalence of infection, although the subsequent laboratory confirmation adopted in the present study increased the diagnostic specificity of the final case definition. Histopathological examination was not performed. Although it was beyond the scope of the present study, histopathology could have provided additional information on the nature of the lesions that were negative for *Echinococcus* spp. by PCR.

Anatomopathological and molecular diagnostics also present intrinsic limitations. Advanced cyst degeneration can obscure the typical germinal and laminated layers, making histological identification challenging or impossible [24]. Similarly, PCR sensitivity may be reduced in sterile or heavily degenerated cysts due to low parasite DNA content, the presence of PCR inhibitors, or DNA degradation associated with chronic inflammatory responses [24].

Results of the prospective study emphasize the diagnostic limitations of routine postmortem inspection in hypoendemic settings and highlight the need for complementary diagnostic approaches to avoid misclassification and unnecessary control measures [25]. Integrating molecular diagnostics into routine surveillance would improve diagnostic accuracy and reduce misclassification. Moreover, the study findings suggest the implementation of standardized guidelines for the macroscopic classification of cystic lesions detected at slaughter, particularly in hypoendemic areas. Such an approach, as promoted by the Italian National Parasitological Map [26], could improve the consistency of meat inspection findings and reduce false‐positive diagnoses based solely on gross morphology avoiding unnecessary notifications in areas where CE transmission is minimal or absent [27, 28].

This study has several limitations. First, it was based on an abattoir survey, and therefore, the study population may not be fully representative of the cattle population in the study area, as slaughterhouse‐based surveys are inherently subject to selection bias. Second, the relatively small number of hydatid cysts analyzed reflects the low endemicity of CE in the study area and may have limited the statistical power of the study. Nevertheless, this was also one of the strengths of the study, as it allowed the evaluation of diagnostic challenges associated with CE surveillance in a hypoendemic setting. Moreover, data on farm management practices and dog management were not available in the present study, precluding the assessment of these factors as potential risk determinants. However, all cattle included in the study originated from intensive dairy farms located in Lombardy and Emilia‐Romagna. Unlike traditional pastoral production systems, where close contact between livestock, shepherd dogs, and home slaughter practices facilitates the transmission of *Echinococcus granulosus*, intensive dairy production systems are generally considered less conducive to maintaining the parasite’s life cycle. Therefore, the occurrence of CE observed in the present study may reflect occasional exposure to infected farm or stray dogs and/or inadequate disposal of infected offal, rather than routine farm management practices [14]. Further epidemiological studies incorporating farm‐level management data, together with information on dog ownership and deworming practices, are needed to clarify the transmission pathways of CE in intensive dairy production systems.

CE remains a disease of major public health importance globally, but its epidemiology varies substantially across regions. In hypoendemic areas, a One Health approach should prioritize targeted surveillance, confirmatory diagnostics, and risk‐based interventions rather than blanket control measures [29, 30].

## 5. Conclusion

In conclusion, this study demonstrates that abattoir surveillance alone may overestimate the prevalence of CE in cattle in hypoendemic areas. The present findings indicate that no molecularly confirmed cases of CE were detected among the cattle examined during the study period. These results are consistent with the hypoendemic epidemiological context of northern Italy; however, further studies including multiple slaughterhouses, investigations in definitive hosts, and farm‐level epidemiological data are needed. These findings emphasize the importance of incorporating confirmatory diagnostic methods into surveillance programs to improve diagnostic accuracy, avoid misclassification, and prevent overestimation of disease prevalence. In hypoendemic contexts, surveillance strategies should therefore adopt a risk‐based and integrated approach, combining slaughterhouse inspection with targeted laboratory diagnostics to support evidence‐based control policies and optimize resource allocation.

## Author Contributions

**Laura L. Kramer:** conceptualization (lead), supervision (lead), project administration (lead), funding acquisition (lead), validation (supporting), writing–original draft preparation (supporting), writing–review and editing (equal). **M. Genchi:** conceptualization (lead), supervision (lead), project administration (lead), funding acquisition (lead), validation (supporting), resources (lead), writing–review and editing (equal). **M. Rega:** methodology (lead), software (lead), formal analysis (lead), investigation (lead), data curation (lead), visualization (lead), writing–original draft preparation (lead), writing–review and editing (equal). **M. Fozzer:** conceptualization (lead), supervision (lead), project administration (supporting), methodology (lead), software (lead), formal analysis (lead), investigation (lead), data curation (lead), visualization (lead), writing–review and editing (equal). **A. Vismarra:** methodology (lead), software (supporting), formal analysis (supporting), investigation (supporting), data curation (supporting), visualization (supporting), writing–review and editing (equal). **C. Loiudice:** validation (supporting), resources (lead), writing–review and editing (equal). **A. Parrinello:** validation (supporting), resources (lead), writing–review and editing (equal). **M. Conter:** visualization (supporting), writing–review and editing (equal). **G. Pupillo:** Methodology (supporting), software (supporting), formal analysis (supporting), investigation (supporting), data curation (supporting), visualization (supporting), writing–review and editing (equal).

## Funding

The authors have nothing to report. Open access publishing facilitated by Universita degli Studi di Parma, as part of the Wiley ‐ CRUI‐CARE agreement.

## Ethics Statement

Access to slaughterhouse records and sample collection were authorized by the Local Health Authority and conducted within the framework of the Specialization School in Inspection of Food of Animal Origin, University of Parma. The study was based on data collected during routine official controls, and all information was anonymized before analysis.

## Consent

The authors have nothing to report.

## Conflicts of Interest

The authors declare no conflicts of interest.

## Data Availability

All data generated or analyzed during this study are included in this published article.
